# Evaluation of the Relationship Between Preferential Looking Testing and Visual Evoked Potentials as a Biomarker of Cerebral Visual Impairment

**DOI:** 10.3389/fnhum.2021.769259

**Published:** 2021-10-27

**Authors:** Sruti Raja, Batool Sahar Emadi, Eric D. Gaier, Ryan A. Gise, Anne B. Fulton, Gena Heidary

**Affiliations:** ^1^Department of Ophthalmology, University of Florida College of Medicine, Gainesville, FL, United States; ^2^Department of Ophthalmology, Harvard Medical School and Boston Children’s Hospital, Boston, MA, United States; ^3^Picower Institute for Learning and Memory, Massachusetts Institute of Technology, Cambridge, MA, United States

**Keywords:** cerebral visual impairment, CVI, visual acuity, preferential looking, visual evoked potential

## Abstract

Cerebral visual impairment (CVI) is a leading cause of visual impairment in children in developed countries, but diagnostic tools to detect CVI are limited. We sought to analyze the visual acuity of children with CVI as assessed by visual evoked potentials (VEPs) and preferential looking test (PLT) to determine whether the relationship between the visual outcomes on these two testing methods may serve as a biomarker of CVI. We performed a retrospective chart review of patients with a confirmed diagnosis of CVI and at least one ophthalmological assessment with visual acuity measured by VEP and PLT. Of the 218 patients included in the study, the most common condition associated with CVI was an underlying genetic disorder (36%, 79/218). Treatment for seizures occurred in the majority of the entire cohort of patients (80%, 175/218). Ophthalmic comorbidities included retinal disease in 23 patients, optic nerve disease in 68 patients, nystagmus in 78 patients, and strabismus in 176 patients. When assessed by either VEP or PLT, visual acuity in children with CVI fell below expected norms. At initial and final presentations, VEP acuity exceeded PLT acuity by one or more octaves, and this difference was greater than expected compared with normal visual development. We propose utilizing this quantifiable disparity between VEP and PLT as a biomarker of CVI.

## Introduction

Cerebral visual impairment (CVI) is a leading cause of visual impairment in children in developed countries (Afshari et al., 2001; Good et al., 2001; Hoyt, 2003). CVI has been defined as “verifiable visual dysfunction which cannot be attributed to disorders of the anterior visual pathways or any potentially co-occurring ocular impairment” (Sakki et al., 2018), and recent studies have sought to further clarify the neuroanatomic basis of CVI (Merabet et al., 2017). The number of conditions associated with the development of CVI are myriad and include perinatal hypoxic ischemic injury, genetic disorders, metabolic disorders, infection, trauma, and epilepsy (Merabet et al., 2017). CVI reflects brain-based visual dysfunction with often normal ocular structures (Lim et al., 2005). Thus, the diagnosis of CVI is often difficult to establish and therefore, biomarkers of CVI are needed.

The purpose of this study was to compare visual measures in children with CVI using two reliable and validated methods of testing grating visual acuity: visual evoked potential (VEP) and preferential looking test (PLT; Birch and Bane, 1991; Bane and Birch, 1992; Good, 2001). The VEP is an electrophysiologic measure of coordinated neural activity elicited by a visual grating stimulus and reflects the integrity of the visual pathway from the retina to the visual cortex (Good et al., 1994). PLT utilizes a forced choice method of visual acuity assessment and relies on higher order visual function. PLT requires that the child not only recognize the stimulus but also process and act on this detection by making a saccadic eye movement toward the stimulus (Hamilton et al., 2021). Based on this inherent difference in methodology, we hypothesize that children with CVI would exhibit reduced visual acuity measures. Further that the disparity between VEP and PLT measures may reflect higher-order cerebral dysfunction and thereby serve as a quantifiable biomarker of CVI.

## Materials and Methods

This study was approved by the Boston Children’s Hospital (BCH) Institutional Review Board and conducted in compliance with the Health insurance Portability and Accountability Act and the tenets of the Declaration of Helsinki. Patients were included by waiver of consent for retrospective data collection.

A retrospective chart review of patient records from January 2005 through December 31, 2020 was performed of patients 18 years and younger at the first visit seen for at least one eye examination in the Department of Ophthalmology at BCH who had undergone VEP (CPT code 95930) and who were coded as having CVI (ICD 10 H47 619, 611, 612 or ICD 9 377.75). Charts were reviewed in detail, and the following inclusion criteria were applied: (1) confirmation of the diagnosis of CVI based on clinical history and examination findings, (2) at least one examination in which VEP and PLT were performed on the same visit. All children included in the study met the proposed criteria for diagnosis of CVI defined as “verifiable visual dysfunction which cannot be attributed to disorders of the anterior visual pathways or any potentially co-occurring ocular impairment” (Sakki et al., 2018).

Data including patient demographic, ophthalmic data, and medical history regarding conditions commonly associated with CVI were collected. Binocular test results for VEP and PLT were recorded for initial and most recent visits, when multiple visual assessments had been made for the same patient. The presence of retinal disease, optic nerve disease, nystagmus, and strabismus was recorded for each patient.

### Visual Acuity Procedures

Visual acuity assessment by PLT was performed according to standard clinical practice, and as previously described in detail (Teller et al., 1986; Lim et al., 2005).

Details of the sweep visual evoked potential (sVEP) procedure have been previously described (Fulton et al., 2005; Lim et al., 2005). In brief, sVEP were recorded using the NUDiva system (Norcia and Tyler, 1985; Taylor and McCulloch, 1992). Electrodes were located as follows: the reference electrode was placed at the vertex, a ground electrode was placed on the forehead, additional placements were 3 cm above the inion (Oz) and 3 cm to the left (O1) and right (O2) (Lim et al., 2005). Stimuli consisted of a high-contrast (80%) vertical square-wave grating which was alternating at a frequency of 5.5 Hz with an average luminance of 76 cd/m^2^. Gratings were swept from low to high spatial frequency during 10-s trials. The mean of 5 or more sweeps was utilized to estimate visual acuity with a linear extrapolation method determining the spatial frequency that produced a 0 μV response (Lim et al., 2005).

### Data Analysis

Visual acuity measures were compared to published, normative data using PLT and sVEP in pediatric patients as a function of age (Orel-Bixler, 1989; Birch, 2006; Leone et al., 2014). PLT acuity was compared to sVEP acuity within patients on a log_2_-based or octave scale and their relationship was assessed by determining the Pearson correlation coefficient and a paired student’s *t*-test. Each octave represents a doubling of spatial frequency on the grating acuity (Lim et al., 2005). Longitudinal visual acuity measures in patients with more than one visual acuity assessment evaluating initial and most recent visits were compared using a paired student’s *t*-test. In all cases, *p* < 0.05 was considered the threshold for statistical significance.

## Results

The charts of 311 patients who were coded as CVI with a VEP were reviewed. Patients who were older than 18 years at their first visit, in whom a diagnosis of CVI could not be confirmed, and for whom both visual acuity measures were not obtained at a single eye exam were excluded. In total, 218 patients (104 females, 114 males) met inclusion criteria for the study.

### Cerebral Visual Impairment Phenotype

The clinical characteristics of this cohort are summarized in Table 1. Of the 218 patients meeting inclusion criteria, underlying genetic disease was the most frequent medical condition, affecting 79 patients (36%). Genetic abnormalities included 8 patients (4%) with chromosomal abnormalities; in addition, genetic diagnoses were heterogeneous and included those associated with neurodevelopmental abnormalities and seizures such as Rett syndrome and GRIN associated disorders. Additional comorbidities included a history of prematurity, prematurity with periventricular leukomalacia (PVL) confirmed with magnetic resonance imaging of the brain or computed tomography of the head, congenital brain malformations, perinatal insult, traumatic brain injury during the first year of life, and neurodegenerative disease. The category denoted as “Other” comprised of patients with complex neurological, developmental, and medical histories without a definitive diagnosis or one which did not fall into the categories listed above. Among the cohort, 175 (80%) were treated with continuous medication and followed by neurology for a seizure disorder.

**TABLE 1:** Clinical characteristics of cerebral visual impairment (CVI) cohort.

	***N* = 218**	**(%)**
**Sex, Female**	104	48
**Associated Primary Medical Comorbidity**		
Prematurity	16	7
Prematurity with Periventricular Leukomalacia	10	5
Genetic Disorder	79	36
Congenital Brain Malformation	36	17
Hypoxic Ischemic Encephalopathy	27	12
Traumatic Brain Injury	11	5
Perinatal Meningitis/Encephalitis	7	3
Perinatal Stroke	8	4
Neurodegenerative Disease	7	3
Congenital Cytomegalovirus or Toxoplasmosis Infection	4	2
Other	13	6
**Treatment for Seizure Disorder**	175	80
**Cerebral Palsy**	61	28

### Ophthalmological Phenotype

The ophthalmological profile is outlined in Table 2. The median age at first assessment of visual acuity assessment was 1 year 8 months (range 2 months – 17 years 10 months). 152 patients had longitudinal assessments of visual acuity measured by both PLT and VEP methods. The median age at most recent assessment of visual acuity was 6 years and 1 months (range 11 months – 20 years). A subset of patients had ocular abnormalities which included 23 patients with retinal disease and 68 patients with optic nerve disease including optic nerve pallor and hypoplasia. Nystagmus was observed in 78 patients and strabismus was observed in 176 patients; among children with strabismus, the majority had an underlying exotropia.

**TABLE 2:** Ophthalmological characterization of CVI patients.

**Age at Testing**	**Median (year:months)**	**Range (year:months)**
Age at first visual acuity assessment (*N* = 218)	1:8	0:2–17:10
Age at recent visual acuity assessment (*N* = 152)	6:1	0:11–20:0
**Ophthalmological findings**	N	(%)
Diagnosis of CVI	218/218	100
Nystagmus	78/218	36
Strabismus	176/218	81
Esotropia	35/176	19.9
Exotropia	140/176	79.5
Hypertropia	1/176	0.6
Retinal disease	23/218	11
Optic nerve disease	68/218	31
**Visual Acuity**	N	Mean (cycles per degree)
PL acuity at first assessment	218/218	2.0
sVEP acuity at first assessment	218/218	5.5
PL acuity at recent assessment	152/218	2.8
sVEP acuity at recent assessment	152/218	11.2

### Visual Acuity

**FIGURE 1:**
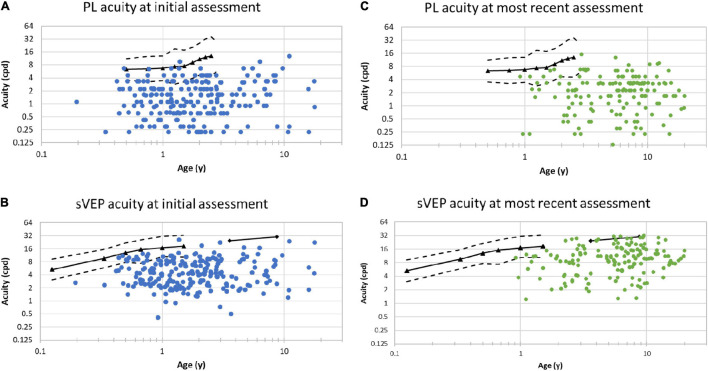
Binocular visual acuities assessed by two different methods at two distinct time points. Acuity (ordinate) is on a log_2_ scale and age (abscissa) is on a log_10_ scale; cpd indicates cycles per degree. **(A)** Preferential looking acuities at first clinical visit. **(B)** Sweep visual evoked potential (sVEP) acuities at first clinical visit. **(C)** Preferential looking acuities at most recent clinical visit. **(D)** sVEP acuities at most recent clinical visit. **(A,C)** Mean normal preferential looking acuity (black triangles) and the 95% limits of normal acuity (dashed lines) based on published results in age matched controls (Leone et al., 2014). **(B,D)** Mean sVEP acuities in typically developing children (black triangles Birch, 2006 and diamonds Orel-Bixler, 1989) based on published findings; the dashed lines represent the 95% limits of normal acuity.

Visual acuity measures for binocular acuity are plotted as a function of age in Figure 1. Overall visual acuity measures were worse for patients with CVI compared to age-matched, published normative data (Leone et al., 2014). The overall mean preferential looking acuity in patients with CVI was 2 cpd (range 0.2–12.8 cpd). The mean preferential looking acuity of 1-year old infants in this cohort with CVI was 1.7 cpd (*n* = 11) compared with a mean of 6.7 cpd (Leone et al., 2014). Only two patients exceeded the mean PL acuity compared with age matched normative data. The majority of patients fell below the lower 95% prediction limit compared with the normative group (Leone et al., 2014). Longitudinal normative data have been published using PLT (Leone et al., 2014). At 33 months old, in the CVI study cohort, mean visual acuity was 2.2 cpd (*n* = 8) compared with a mean preferential acuity of 12.6 cpd in a population with normal development (Leone et al., 2014).

In the CVI study cohort, the overall mean sVEP acuity was 5.5 cpd (range 0.4–26 cpd). When focused on the subgroup of 1-year old patients in this cohort, the mean sVEP acuity was 4.6 cpd (*n* = 11) compared with a mean sVEP acuity of 16.9 cpd in normal visual development (Birch, 2006). At 33 months, in the CVI study cohort, the mean sVEP acuity was 9.2 cpd (*n* = 8). In comparison to age matched normative data, the sVEP acuity for the CVI study cohort fell below the lower 95% prediction limit (Orel-Bixler, 1989; Birch, 2006).

Of the 218 patients evaluated, 152 (70%) had additional measures of visual acuity by PLT and sVEP on subsequent eye visits. The results of acuities assessed by PL and sVEP methods at the patient’s most recent visit are seen in Figures 1C,D, respectively. At the most recent visit, visual acuity continued to be subnormal when tested by either method. At the final visit, mean PL acuity was 2.8 cpd (range 0.13–15 cpd) and mean sVEP acuity was 11.2 cpd (range 1.2–32 cpd). Compared with the first visit, there was a demonstrable improvement in vision which was statistically significant both for PLT (*p* = 0.004) and also for sVEP (*p* < 0.001).

**FIGURE 2:**
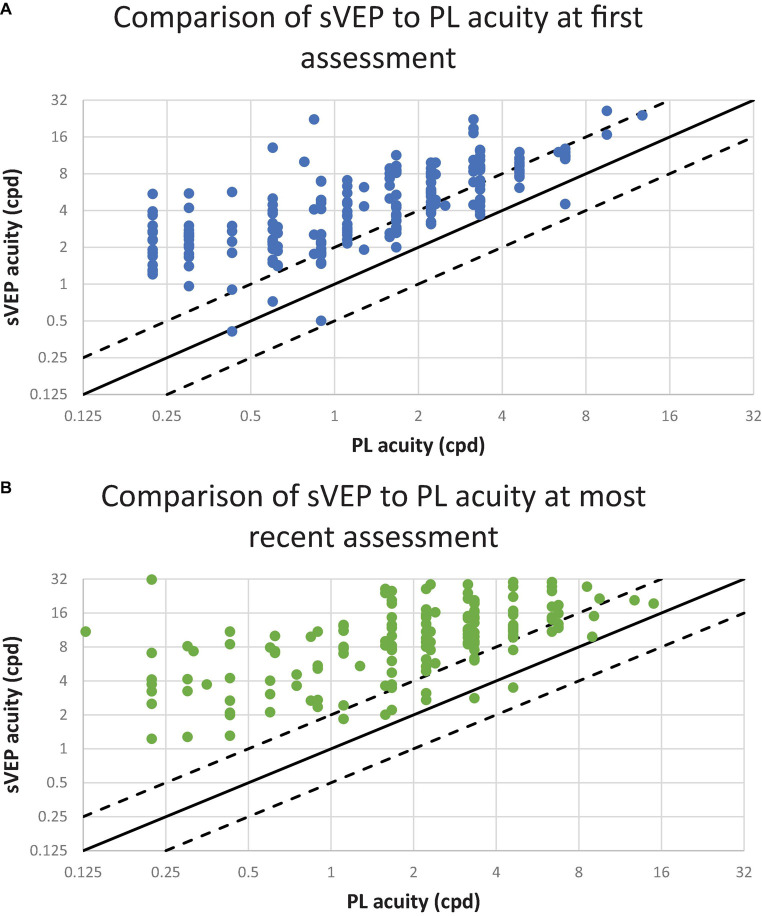
The relationship between preferential looking (PL) and visual evoked potential (VEP) acuities. Each point represents one patient. The diagonal lines have a slope of 1.0. The solid line represents PL and sVEP acuity values in perfect agreement. The dashed lines 1 octave above and below the solid line. The ordinate and the abscissa are a log_2_ scale; cpd indicates cycles per degree. **(A)** Visual acuity assessments conducted at the patient’s first visit. The results from 225 patients are plotted. **(B)** Visual acuity assessments conducted at the patient’s most recent visit. The results from 156 patients are plotted.

Figure 2 demonstrates the relationship between PLT acuity and sVEP acuity. Nearly all data points are located above the line of unity, illustrating that visual outcome measured by sVEP exceeds that measured by PLT. Furthermore, the majority of data points lie above the dashed line signifying that sVEP exceeds PL acuity by 1 or more octaves at the first (Figure 2A) and most recent (Figure 2B) assessment. At first assessment of visual acuity, a moderate correlation was found between preferential looking and sVEP acuities (Pearson correlation, *r* = 0.72, *p* < 0.001). This result was also observed at the most recent assessment of visual acuity (Pearson correlation, *r* = 0.48, *p* < 0.001).

## Discussion

Herein, we present binocular visual acuity outcomes in pediatric patients with CVI. To our knowledge, this is the largest study of visual outcome measures in children with CVI. We found that in comparison to normal development, patients with CVI had worse visual acuities when assessed by either method. In most cases, sVEP acuity was better than PL acuity. Longitudinally, although visual acuity did improve, children with CVI continued to demonstrate subnormal vision for age.

The main objective of this study was to assess the relationship between PL and sVEP acuities in patients with CVI. For most patients, we found that sVEP acuities exceeded PL acuities by more than one octave at initial and last assessment. Although in early visual development, VEP acuity is expected to mature more rapidly than PL acuity, this disparity is thought to be on the order of 1 octave in normal development; with visual maturation, the gap between the two measures is anticipated to narrow (Lim et al., 2005). In contrast, we found in children with CVI, the difference between VEP acuity and PL acuity is larger than expected compared with normal development and this continued to be present in spite of visual maturation. Beyond subnormal vision for age by both measures, we hypothesize that both the extent and the persistence of this difference between PLT and sVEP acuity at any given age in the cohort may be a potential indicator of CVI and this warrants further investigation. This profile seen in the CVI study cohort is distinct from normal visual development in which unequal performance on these measures in early visual development (sVEP exceeding PLT) should narrow to more symmetric performance once vision matures normally (Lim et al., 2005). Although it is possible that the comorbidity of cerebral palsy (CP), which occurred in 28% of this cohort, may contribute to difficulty in performance on PLT with respect to oculomotor function, the disparity between the two visual acuity measures occurred in patients with or without CP. Therefore, this disparity between the two measures cannot be explained by CP alone, but likely reflects more global dysfunction in CVI in which there are deficits in visual perception, visual attention, and oculomotor function (Salati et al., 2002).

The results of this study are consistent with previously published reports on the relationship between PL and sVEP acuity in the context of CVI (Good, 2001; Lim et al., 2005; Watson et al., 2010; Olson et al., 2021). In one study of 19 patients with CVI associated with a history hypoxic-ischemic encephalopathy, the authors found the discrepancy between VEP and PL acuity to be greater than one octave in more than half of the patients (Lim et al., 2005). More recently, a similar disparity between PL and sVEP acuities were described in 11 patients with a genetically confirmed seizure disorder CDKL5 and CVI (Olson et al., 2021). Our study expands on the generalizability of these findings with visual acuity measures from over 200 patients and the inclusion of patients with a diverse range of medical conditions associated with CVI. The study was limited by the requirement for measurement of VEP and PLT at the same visit which may have biased the cohort toward those patients whose visual dysfunction was more profound. Therefore, the applicability of our findings to patients with visual perceptual disorders who may perform optotype acuity testing is uncertain and warrants further investigation.

Diagnosing CVI can be challenging due to its variability in clinical presentation, the presence of additional comorbidities, and the fact that CVI reflects brain-based visual dysfunction. This highlights the need to identify a quantifiable visual profile or visual biomarker of disease to aid in establishing the diagnosis of CVI. In this study, we have shown that visual acuity by PLT and sVEP is consistently lower in patients with CVI across a range of etiologies. Further, the gap in PL and sVEP acuities exceeds what is expected in normal development. Future studies that evaluate the relationship between this potential indicator of CVI and other aspects of functional vision, neuroimaging findings, or cognitive and motor development will allow for a more thorough characterization of the clinical phenotype of CVI and yield insight into areas of accommodation and support for children impacted by this condition.

## Data Availability Statement

The datasets presented in this article are not readily available because they contain identifiable information. Requests to access the datasets should be directed to GH, gena.heidary@childrens.harvard.edu.

## Ethics Statement

The studies involving human participants were reviewed and approved by the Boston Children’s Hospital (BCH) Institutional Review Board and conducted in compliance with the Health insurance Portability and Accountability Act and the tenets of the Declaration of Helsinki. Patients were included by waiver of consent for retrospective data collection. Written informed consent from the participants’ legal guardian/next of kin was not required to participate in this study in accordance with the national legislation and the institutional requirements.

## Author Contributions

GH conceptualized the study, analyzed the results, contributed to manuscript preparation, and approval for publication. SR conducted the chart review and analyzed the results. BE conducted the chart review. All authors contributed to the preparation of the manuscript and approval for publication.

## Conflict of Interest

The authors declare that the research was conducted in the absence of any commercial or financial relationships that could be construed as a potential conflict of interest.

## Publisher’s Note

All claims expressed in this article are solely those of the authors and do not necessarily represent those of their affiliated organizations, or those of the publisher, the editors and the reviewers. Any product that may be evaluated in this article, or claim that may be made by its manufacturer, is not guaranteed or endorsed by the publisher.
